# The influence of astragalus polysaccharide and β-elemene on LX-2 cell growth, apoptosis and activation

**DOI:** 10.1186/s12876-014-0224-8

**Published:** 2014-12-31

**Authors:** Jin Zheng, Li-tian Ma, Qin-you Ren, Lu Li, Yi Zhang, Heng-jun Shi, Yi Liu, Cheng-hua Li, Yong-qi Dou, Shao-dan Li, Hui Zhang, Ming-hui Yang

**Affiliations:** Department of Traditional Chinese Medicine, General Hospital of People’s Liberation Army, Beijing, 100853 China; Department of Traditional Chinese Medicine, Tangdu Hospital, The Fourth Military Medical University, Xi’an, 710038 China; Department of Oncology, Shaanxi province Hospital of Traditional Chinese Medicine, Xi’an, 710003 China

**Keywords:** Astragalus polysaccharide, β-elemene, Hepatic stellate cells

## Abstract

**Background:**

Activated hepatic stellate cells are the main source of excessive collagen deposition in liver fibrosis. Here we report the inhibitory effects of the combinational treatment of two natural products, astragalus polysaccharide (APS) and β-elemene (ELE) on the activation of human liver hepatic stellate cell line LX-2 cells.

**Methods:**

Cultured LX-2 cells were treated with different concentrations of APS or ELE for 24 or 48 hours. Cell viability/apoptosis was measured by MTT assay and Annexin V/PI staining , activation related genes including α-SMA and CD44 expressions were measured by real-time PCR and western blot respectively.

**Results:**

The majority of LX-2 cells showed morphological change in the presence of APS or ELE for 24 hours. Treatment with APS + ELE for 24 or 48 hours significantly inhabited the cell proliferation compared with APS or ELE treatment alone on LX-2 cells. APS + ELE may block the up-regulation of α-SMA and CD44 both in mRNA and protein levels through TGF-β pathway in LX-2 cells.

**Conclusion:**

APS or ELE treatment alone on LX-2 cells could inhibit cell proliferation and induce apoptosis. The combinational treatment using APS + ELE significantly increased the killing efficiency on LX-2 cells. α-SMA and CD44 expressions was inhibited upon APS + ELE treatment through TGF-β pathway in LX-2 cells. The results indicated a novel treatment using natural products for liver diseases with anti-fibrotic effect.

## Background

Liver fibrosis is characterized by the accumulation of extracellular matrix proteins like collagen. It occurs in many types of chronic liver diseases induced by viral infection, alcohol intake and high-fat diet. Hepatic stellate cells (HSCs) were identified as the main cell type that produce collagen in liver fibrosis when these cells were activated [1]. So, looking for drugs which can prevent the activation or induce apoptosis of HSCs will be very helpful for treating liver fibrosis.

LX-2 cell line is a well characterized human HSC cells line, it is similar to that of “activated” HSC cells in vivo. LX-2 cells retain the key features of cytokine signaling, retinoid metabolism, and fibrogenesis of activated HSCs [2]. So, it is widely used for the studies of pathogenesis of liver fibrosis and developing new approaches for liver fibrosis treatment [3-6].

It was believed that liver fibrosis was irreversible previously, however, recent studies suggested that liver fibrosis was reversible. Several mechanisms were involved in the process:1. Kupffer cells (KC) derived cytokines can promote HSC activation in the development of liver fibrosis, however, TNF-related apoptosis-inducing ligand (TRAIL) expressed by KCs can induce apoptosis of HSCs in fibrosis resolution [7]. 2. Nature killer (NK) cells may directly kill activated HSCs and promote liver fibrosis resolution [8].

Recent studies indicated that astragalus polysaccharide (APS) and β-elemene (ELE) showed prominent anti-fibrotic effect in treating liver fibrosis [9-12]. The role of APS, the major bioactive component of Astragalus mongholicus, in immune regulation has been well studied. Specifically, APS may regulate the KC cell function and promote the cytotoxicity of NK cells [13,14]. These results suggest that APS may be helpful in the resolution of liver fibrosis by regulating the function of immune cells in the liver. ELE is a volatile terpene found in many plants such as celery and mint. ELE has been widely used in treating liver fibrosis and liver cancer in China [15]. It can strongly inhibit the production of extracellular matrix (ECM) by activated HSCs. So, we believe that the combination of ELE and APS may not only block the production of ECM but also promote the resolution of liver fibrosis by regulating the function of immune cells in the liver which will be an ideal treatment for liver fibrosis. In this study, we employed the LX-2 cell line to investigate the anti-fibrotic effects of APS and ELE and the possible underlying mechanisms.

## Methods

### Materials

APS was obtained from Cinorch Pharmaceutical (Tianjin, China). ELE was obtained from Sigma .Human HSC LX-2 cell line was obtained from The Fourth Military Medical University. Dulbecco’s modified Eagle’s medium (DMEM), penicillin/streptomycin and glutamine were purchased from Gibco (Foster City, CA, USA), fetal bovine serum (FBS) was purchased from Sijiqing (Hangzhou, China). TGF-β1 was purchased from Peprotech (Rocky Hill, NJ, USA).

### Cell culture

LX-2 cells were cultured in DMEM supplemented with 10% FBS, 100 IU/ml penicillin/streptomycin and 1% glutamine. Cultures were incubated at 37 degree in a humidified atmosphere of 5% CO_2_, and the medium was changed every day.

### Measurement of cell viability

Cell viability was determined by MTT assay. A total of 1X10^4^ cells were seeded in 96 well plates, When the confluence reached to 80% ~ 90%, medium was changed, different concentrations of APS, ELE or combination of APS and ELE (APS + ELE) were added into the medium. 24 or 48 hours later, 20 μl 5 mg/ml MTT was added into wells and incubated for 4 hours. After adding 150 μl DMSO, the optical density was measured at 490 nm.

### Annexin V/PI assays for apoptosis

Per manufacturer’s protocol (ebioscience, San Diego, CA, USA),cells were stained with Annexin V–FITC and PI, and evaluated for apoptosis by flow cytometry. Briefly, 1 X 10^6^ cells were washed twice with phosphate-buffered saline (PBS), and stained with 5 μl of Annexin V–FITC and 10 μl of PI (5 μg/ml) in 1X binding buffer (10 mM HEPES, pH 7.4, 140 mM NaOH, 2.5 mM CaCl2) for 15 min at room temperature in the dark. The apoptotic cells were determined using a BD FACSAria (Mansfield, MA, USA). Both early apoptotic (Annexin V-positive, PI-negative), late apoptotic (Annexin V-positive and PI-positive) and dead (Annexin V-negative and PI-positive) cells were included in cell death determinations.

### Real-time quantitative PCR

Total RNA was isolated from cells using Trizol Reagent (Invitrogen) and quantified. cDNA was synthesized from 1μg of RNA using High Capacity cDNA Reverse Transcription Kit (Applied Biosystems, Foster City, CA, USA). The cDNA was used as templates for realtime quantitative PCR using the Prism 7500 real-time PCR instrument with SYBR Green PCR Master Mix (Applied Biosystems). The primer sequences were: β-actin :5′-GATCATTGCTCCTCCTGAGC-3′, 5′-TGTGGACTTGGGAGAGGACT-3′; α-SMA:5′-GACAATGGCTCTGGGCTCTGTAA-3′, 5′-CTGTGCTTCGTCACCCACGTA-3′; CD44: 5′-GCTTCCAGAGTTACGCCCTTGA-3′, 5′-AACCCTTGCAACATTGCCTGA-3′.

### Western blot analysis

LX-2 cells were lysed in RIPA buffer containing 1% PMSF and protease inhibitor cocktail. The cell lysate was centrifuged at 12000 rpm for 20 min at 4 degree and the supernatant was collected. Protein concentration was determined by using BCA protein assay. A total of 40 μg of cell lyse were separated by SDS-PAGE gel and transferred to NC membranes. The membranes were incubated with anti-CD44s and, anti-α-SMA antibodies (Abcam, Cambridge, MA, USA), anti-β-actin antibody (Santa Cruz, Dallas, TX, USA). After washing 3 times with TBS/T buffer, membranes were incubated with a horseradish peroxidase-conjugated secondary antibodies (Boster, Wuhan, China) for 1 h. The blots were developed with ECL (Pierce) according to the manufacturer’s instruction.

### Statistical analysis

Data was presented as mean ± standard deviation (S.D.) of three independent experiments. The results were analyzed by one-way analysis of variance (ANOVA) or Student *t* test using SPSS17.0 software. Differences were considered as being significant at P < 0.05.

## Results

### The inhibitory effects of APS and ELE on LX-2 cells

In control group, LX-2 cells showed typical HSC morphology with extended dendrites. 24 hours after APS treatment, cell fusion was observed. In addition, some of the cells showed a round cell shape with decreased dendrites and increased vesicular structures. 48 hours later, most of the cells turned to an enlarged round shape and increased vesicular structures. Enlarged round shape and increased vesicular structures were also observed in ELE group at 48 hours after treatment. Similar morphology was be observed as early as 24 hours after the combination of both APS and ELE (APS + ELE) treatment.

### The influence of APS and ELE on LX-2 cell proliferation by MTT assay

**Figure 1:**
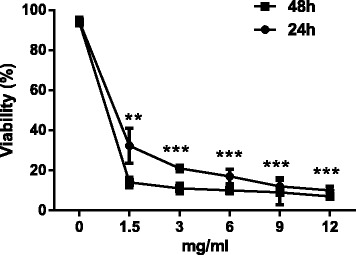
**Effects of APS on the viability of LX-2 cells.** LX-2 cells were treated with different concentration of APS as indicated for 24 or 48 hours. Viability was determined by MTT assay. **P < 0.01, ***P < 0.001 compared to untreated cells at the same time point.

**Figure 2:**
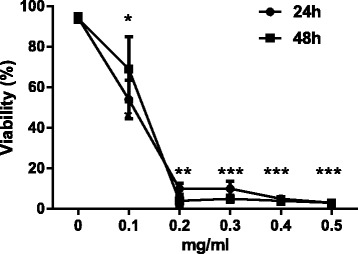
**Effects of ELE on the viability of LX-2 cells.** LX-2 cells were treated with different concentration of ELE as indicated for 24 or 48 hours. Viability was determined by MTT assay *P < 0.05, **P < 0.01, ***P < 0.001 compared to untreated cells at the same time point.

Both APS and ELE could significantly inhibit the cell viability of LX-2 cell in a dose- and time-dependent manner as shown in Figures 1 and 2. We showed that when the concentration of APS was higher than 3 mg/ml, the viability of APS treated cells only slightly decreased when the dose was further increased. So we selected 3 mg/ml in the following experiments. Similarly, 0.2 mg/ml of ELE was selected based on the dose response curve.

**Figure 3:**
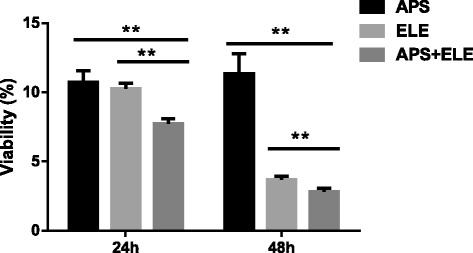
**Effects of APS combined with ELE on the viability of LX-2 cells, LX-2 cells were treated with different concentration of APS, ELE or APS + ELE as indicated for 24 or 48 hours.** Viability was determined by MTT assay **P < 0.01, compared to ELE treated cells in the same time point, **P < 0.01, compared to APS treated cells at the same time point.

We further explored the effects on LX-2 cell viability of combination treatment of both 3 mg/ml APS and 0.2 mg/ml ELE. As shown in Figure 3, we found that the viability determined by MTT assay was 7.6% ± 0.58% in APS + ELE group 24 after treatment, which was significantly lower than in APS alone treatment (10.8 ± 0.34%) or ELE alone (10.6 ± 0.26%) (p < 0.01). 48 hours later, the viability decreased to 2.8% ± 0.16% in APS + ELE group compared with 10.3 ± 0.45% in APS alone or in 3.4 ± 0.12% ELE alone (p < 0.01).

### The effects of APS, ELE alone or APS + ELE treatment on LX-2 cell apoptosis by flow cytometry analysis

**Figure 4:**
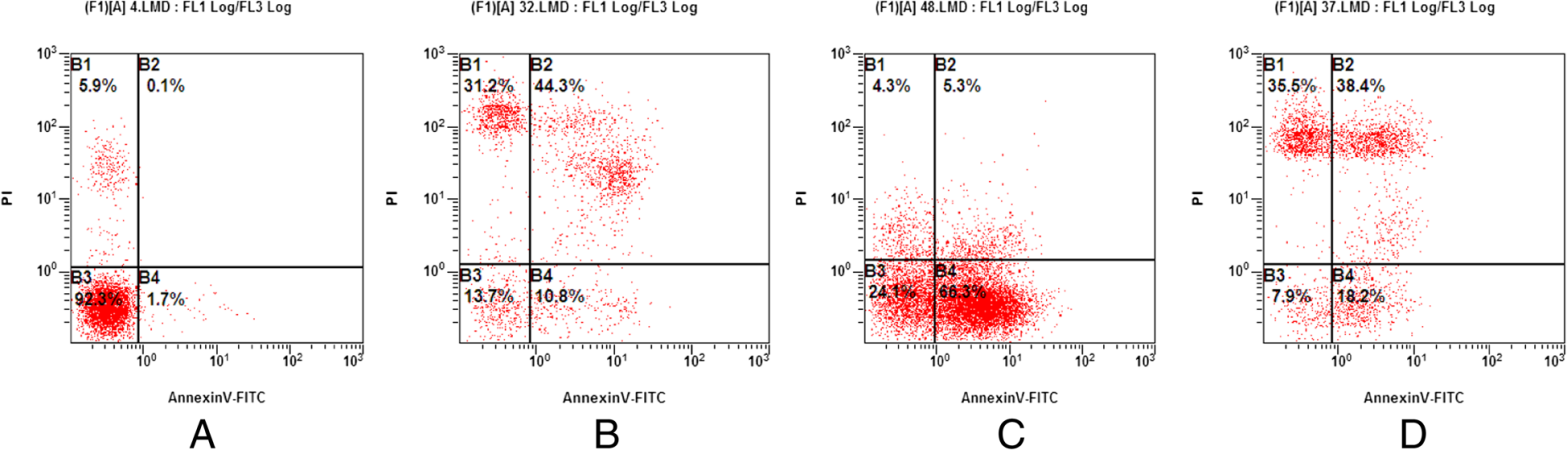
**Apoptosis of LX-2 cells analyzed by flow cytometry 24 hours after treatment of APS, ELE or APS + ELE: A: Control; B: ELE:0.2mg/ml; C: APS:3mg/ml D: Combined.**

The externalization of phosphatidylserine (PS) in living cells was the early events in apoptosis. Annexin V showed a strong and specific affinity for PS and was used here to detect early stage of apoptosis. Annexin V was used in conjunction with propidium iodide (PI) for identification of early and late apoptotic cells. Viable cells with intact membranes exclude PI, whereas the membranes of dead cells are permeable to PI. So, cells that are in early apoptosis were Annexin V positive and PI negative, cells that were in late apoptosis both Annexin V and PI positive and already dead cells were PI single positive. Here, we used Annexin V/PI staining to detect cell apoptosis 24 hour after APS, ELE or APS + ELE treatment. As shown in Figure 4, compared with control group, most cells were in early or late stage of apoptosis with APS and ELE treatment for 24 hours respectively. 24 hours treatment with APS + ELE, the percentage of dead cells (PI single positive) was significantly higher than APS or ELE alone group.

### Combination of APS and ELE blocked the TGF-β1 induced up-regulation of α-SMA and CD44 in both mRNA and protein levels in LX-2 cells

**Figure 5:**
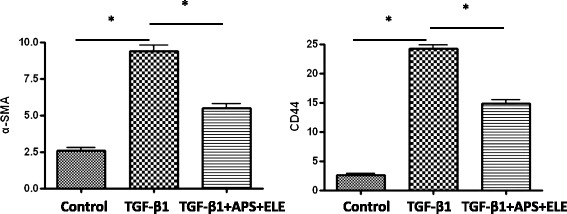
α-SMA and CD44 mRNA of LX-2 cells analyzed by Real-time PCR in Control group, TGF-β group, TGF-β + ELE + APS group (*P < 0.01).

**Figure 6:**
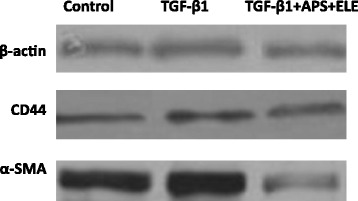
**α-SMA and CD44 protein levels of LX-2 cells analyzed by Western blot in Control group, TGF-β group, TGF-β + ELE + APS group.**

As shown in Figures 5 and 6, both mRNA and protein levels of fibrogenic gene α-SMA and CD44 were significantly increased in LX-2 cells in the presence of TGF-β1 as reported previously. Upon the APS + ELE treatment, the expressions of α-SMA and CD44 induced by TGF-β1 in LX-2 cells were significantly blocked in both mRNA and protein levels (Figures 5 and 6).

**Figure 7:**
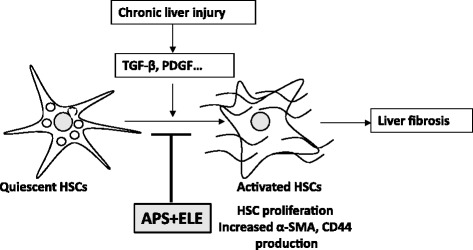
**The mechanism of the anti-fibrosis effect of APA and ELE.**

## Discussion

In current study, we demonstrated that single use of APS or ELE inhibited the growth of LX-2 cells dose- and time- dependently. Combination use of APS and ELE showed significantly increased efficiency in growth inhibition compared with APS or ELE alone. Most of LX-2 cells were in late stage of apoptosis and some of LX-2 cells were already dead with treated with APS + ELE for 24 hours in Annexin V/PI staining. These data suggested that APS can synergistically induce LX-2 cell apoptosis together with ELE.

Recent studies showed that the proliferation and activation of HSCs played a key role in the development of liver fibrosis [1,16]. Targeting the proliferation of HSCs can be considered as novel therapeutic strategy for treating liver fibrosis [17,18]. Drugs which can inhibit the proliferation of HSC cell lines *in vitro* showed promising anti-fibrotic studies effect *in vivo* [12,18,19]. In addition, the resolution of liver fibrosis largely depended on the apoptosis of HSCs [20,21]. Our results indicated that APS + ELE may not only directly block that proliferation of HSC cell line LX-2 cells but also induce LX-2 cells apoptosis, these data suggested that the combinational use of APS and ELE may be also effective in rodent liver fibrosis models or in humans.

The activation of HSCs was characterized by the transformation of quiescent HSCs to extracellular matrix producing phenotype termed myofibroblast [1]. The activation marker α-SMA, which was involved in HSC contraction, was greatly increased in this process of HSC activation [22]. CD44 belongs to transmembrane protein which can bind to hyaluronic acid, fibronectin, laminin and collagen. CD44 was critical in cell motility, tumor development and invasion [23]. Recently, CD44 was reported to be involved in the activation HSCs after liver damage [24]. CD44 was expressed in neutrophils, epithelial cells and endothelial cells and it played important roles in cell adhesion, hyaluronic acid degradation, angiogenesis and cytokine release [25]. CD44 can bind to F-actin through its intracellular ankyrin domain and participate the formation of pseudopodia and cell migration [26]. In the sinusoids, cell connection and signaling was mediated by cell adhesion molecules, including selectin and CD44. The binding of CD44 and its ligands was essential in regulating the adhesion of tumor cell with platelets, leucocytes, sinusoidal endothelial cells and stellate cells, therefore was considered critical in liver metastases [27]. In liver cancer patients with HBV infection and cirrhosis, more attention should be paid at early stage on CD44v expression which was strongly associated with metastases [28]. In addition, it was reported that fibroblast activation protein (FAP) might activate HSCs and increase the expression of MMP2 and CD44 which can enhance the adhesion and invasion of HSCs [29].

## Conclusions

In summary, we used cultured human HSC cell line LX-2 to study the growth inhibition effects of APS and ELE by morphology study, MTT assay and Annexin V/PI staining. We showed that both APS and ELE can inhibit LX-2 cell growth and they may synergistically induce LX-2 cell apoptosis (Figure 7). Furthermore, APS + ELE treatment blocked HSC cell activation marker α-SMA and CD44 expression in both mRNA and protein levels of in LX-2 cells after TGF-β treatment. Our study provided a possible mechanism for the anti-fibrotic effect of APS and ELE, thus a potential new treatment for liver disease wit anti-fibrotic effect.
